# Syringocystadenocarcinoma Papilliferum in a Fifteen-Year-Old Girl: A Case Report and Review of the Literature

**DOI:** 10.1155/2022/8076649

**Published:** 2022-02-03

**Authors:** Jordan N. Halsey, Esteban Fernandez Faith, Suzanna J. Logan, Archana Shenoy, Kathleen M. Schieffer, Catherine E. Cottrell, Anna P. Lillis, Jennifer H. Aldrink, Bhuvana A. Setty, Gregory D. Pearson

**Affiliations:** ^1^Department of Plastic Surgery, Nationwide Children's Hospital, Columbus, OH, USA; ^2^Division of Dermatology, Department of Pediatrics, Nationwide Children's Hospital, Columbus, OH, USA; ^3^The Ohio State University College of Medicine, Columbus, OH, USA; ^4^Department of Pathology and Laboratory Medicine, Nationwide Children's Hospital, Columbus, OH, USA; ^5^The Steve and Cindy Rasmussen Institute for Genomic Medicine, Nationwide Children's Hospital, Columbus, OH, USA; ^6^Department of Radiology, Nationwide Children's Hospital, Columbus, OH, USA; ^7^Department of Surgery, Division of Pediatric Surgery, Nationwide Children's Hospital, Columbus, OH, USA; ^8^Division of Hematology/Oncology/BMT, Nationwide Children's Hospital, Columbus, OH, USA

## Abstract

Syringocystadenocarcinoma papilliferum (SCACP) is a rare malignant neoplasm arising from adnexal tissues and is the malignant complement to the benign neoplasm syringocystadenoma papilliferum (SCAP). SCACP lesions appear as raised nodules or inflammatory plaques and can be associated with SCAP or nevus sebaceous. There have been fewer than 100 described cases of this neoplasm in the literature, and all previously published cases have been described in adults, with the majority occurring in the elderly. We present a case of an adolescent female with a syringocystadenocarcinoma papilliferum arising from a large thigh mass harboring an in-frame alteration in *MAP*2*K*1 along with a brief review of the literature.

## 1. Case Report

**Figure 1:**
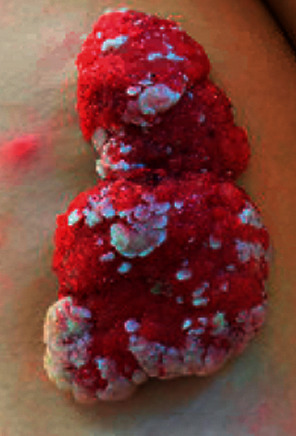
Left posterior thigh pedunculated mass.

A fifteen-year-old female presented to our institution with a large pedunculated posterior thigh mass. According to the patient's mother, the lesion initially appeared as “three small cuts” at birth and grew proportionally with the patient, now appearing hypervascular and measuring 11.5 × 6 × 2 cm in size (Figure 1). There were no reports of pain or bleeding from the lesion, and a review of systems confirmed constitutional symptoms were negative.

**Figure 2:**
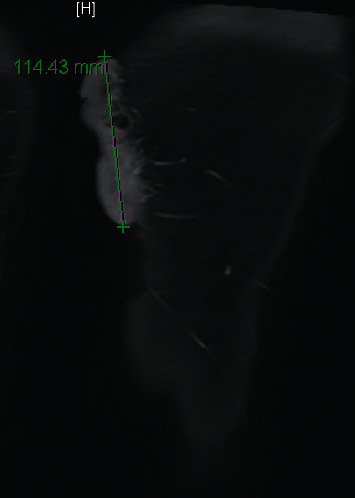
MRI imaging of the left thigh showing posteromedial mass, with arterial blood supply from branches of the deep femoral artery.

There was no prior imaging. An ultrasonographic examination demonstrated two hypoechoic masses with Doppler flow showing arterial and venous enhancement. A subsequent MRI showed the extent of the masses to be 11.9 cm × 6.3 cm × 1.8 cm, extending down to muscle fascia with robust perfusion supplied by perforators of the deep femoral artery (Figure 2). The patient's overall presentation and imaging was discussed at an interdisciplinary vascular malformations conference; the leading diagnosis at that time was syringocystadenoma papilliferum (SCAP). Surgical excision to grossly negative margins was performed with primary wound closure.

**Figure 3:**
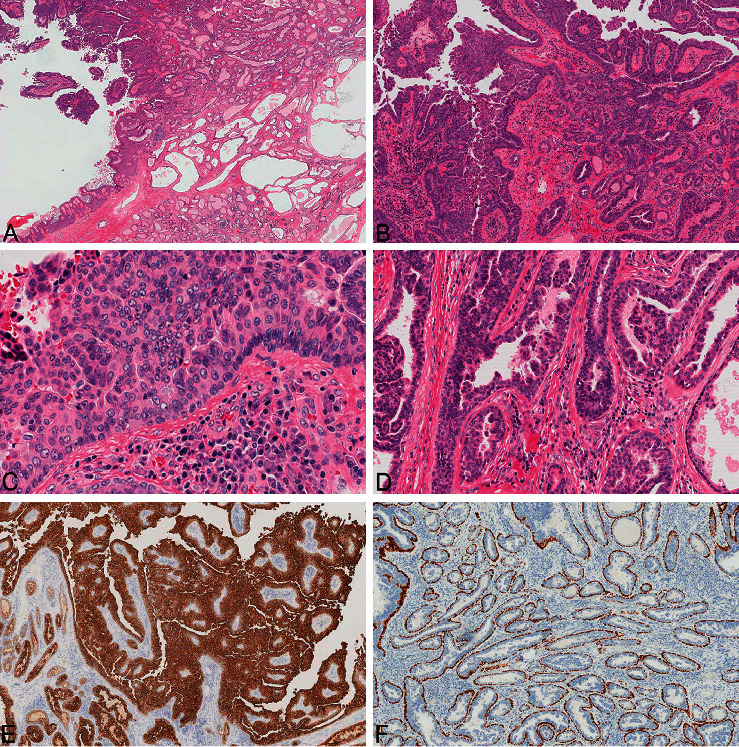
The tumor was composed of both exophytic and underlying infiltrative components, the former showing a papillary pattern similar to syringocystadenoma papilliferum and the latter showing closely spaced tubular glands similar to tubular apocrine adenoma (a). Florid overgrowth of the outer epithelial cells produced complex, maze-like spaces between epithelial cells and cellular tufts off the surface of the tumor (b). The outer epithelial cells showed crowded, overlapping, atypical nuclei with prominent nucleoli with mitotic activity (c). The inner luminal cells of the dermal glands demonstrated bulbous, micropapillary projections (d). CK7 was diffusely expressed by both outer epithelial cells of the papillae and inner luminal cells of the dermal glands (e). P63-positive basal cells bordered the papillae and glands (f).

Microscopic examination revealed an exophytic, papillary lesion with an abrupt transition from the skin surface, overlying a tubular and cystic component diffusely involving the dermis (Figure 3(a)). Papillae were lined by a florid epithelial proliferation forming tufts and maze-like intraepithelial spaces along the lesional surface, with complex glandular spaces within expanded papillary cores (Figure 3(b)). Outer epithelial cells exhibited crowding and disarray and were delimited by a distinct basal cell layer (Figure 3(c)). These lesional cells possessed an eosinophilic cytoplasm, atypical nuclei, fine to optically clear chromatin, and prominent nucleoli (Figure 3(c)). Stromal plasma cell clusters were readily identified within the papillary cores. The deep component extended throughout the dermis and into the subcutaneous tissue, extending to margins of resection. This component was characterized by small tubules lined by a multilayered epithelium with the surface resembling large, dilated glands consistent with an apocrine tubular adenoma component (Figure 3(d)).

Focally, tubules and glands contained intraluminal proteinaceous material with single necrotic cells. Mitotic figures up to 3 per mm^2^ were present throughout the lesion. CK7 immunohistochemistry was diffusely positive in the epithelial cells of the exophytic component and luminal epithelial cells in the dermal component (Figure 3(e)). Based on the florid epithelial proliferation, increased nuclear atypia, and mitotic activity, final diagnosis was SCACP, arising in an SCAP. P63 immunohistochemistry was performed on different blocks and demonstrated basal cells at the periphery of lesional glands and tubules throughout the dermis (Figure 3(f)) as well as in the exophytic component, indicating an *in situ* carcinoma.

**Figure 4:**
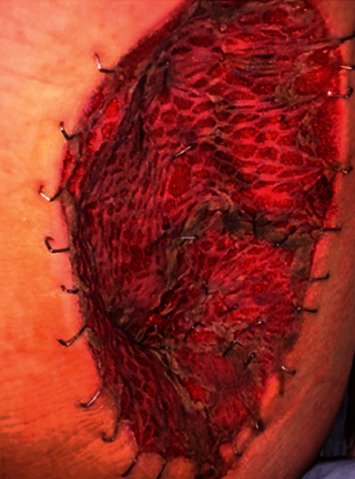
Postoperative image following reresection with margins and skin graft placement.

In response to the SCACP diagnosis, diagnostic imaging of the chest, abdomen, and pelvis with computed tomography (CT) (chest) and magnetic resonance imaging (MRI) (abdomen/pelvis), as well as a full-body PET scan, were obtained. No distant metastases were identified. To complete staging, a left inguinal sentinel lymph node biopsy was performed in conjunction with wide reresection using 2 cm margins. Reresection included all subcutaneous tissue to the level of the semimembranosus muscle fascia in order to ensure complete resection. A skin graft was required to close the large defect on the posterior thigh, which healed well without complication (Figure 4). Although residual SCACP was identified within subcutaneous tissue, surgical margins and sentinel lymph nodes were negative. The patient is now doing well and will be closely followed up for recurrence with routine surveillance.

**Figure 5:**
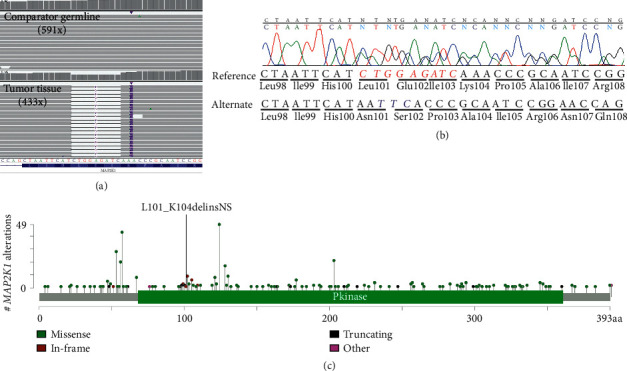
(a) Integrative genomics viewer image of aligned sequencing reads for exon 3 of MAP2K1 derived from enhanced exome sequencing. The top panel shows the comparator germline (peripheral blood) with the absence of the complex indel at an average of 591*x* depth for this region. The bottom panel shows the tumor tissue demonstrating an in-frame complex indel with an average of 433*x* depth for this region. (b) Sanger sequencing chromatogram of the in-frame *MAP*2*K*1 (NM_002755.4) complex indel (c.301_311delinsAATTC:p.Leu101_Lys104delinsAsnSer). The corresponding amino acid sequence is described below the genomic sequence. Italicized reference sequence in red indicates the deletion, and the italicized alternate sequence in blue indicates the insertion. (c) Lollipop plot of *MAP*2*K*1 variants deposited in cBioPortal. The p.Leu101_Lys104delinsAsnSer described in this report has not been previously reported.

Given the rare diagnosis of SCACP, the patient was enrolled on an institutional translational research protocol (see the supplementary materials (available here)). Paired comparator germline and disease-involved enhanced exome sequencing was performed to evaluate for single-nucleotide variants (SNVs), small insertion-deletions (indels), and copy number alterations (CNAs). RNA sequencing of disease-involved tissue was performed to assess for gene fusions, complex structural rearrangements, and outlier gene expression. We did not identify any clearly medically meaningful variation in cancer or other disease-associated genes in the comparator germline. Furthermore, no somatic CNAs, gene fusions, or complex structural rearrangements were identified. The tumor tissue harbored a complex indel in exon 3 of *MAP*2*K*1 (NM_002755.4, encoding MEK1) resulting in an in-frame deletion of codons Leu101-Lys104 (ΔLEIK), replaced with two amino acids (Asn and Ser) (c.301_311delinsAATTC:p.Leu101_Lys104delinsAsnSer) (Figure 5(a)). This in-frame complex indel was confirmed by Sanger sequencing (Figure 5(b)). Although this particular alteration has not been described in the literature, deletions of amino acids encompassed within this alteration, specifically p.Glu102_Ile103del, are described in cancer databases, such as cBioPortal (Figure 5(c)). In-frame deletions constituting part of the *β*3-*α*C loop within the kinase domain (encoded by amino acids 98–104) encompass a class of MAP2K1 variants that demonstrate RAF-independent constitutive signaling due to receptor homodimerization and transphosphorylation.

This patient is the youngest described case of SCACP in the literature, which reports the median age of SCACP to be 63.6 years (reported range 22–93) [1]. These lesions are known to arise from existing SCAP lesions or nevus sebaceous and commonly occur within the head and neck [2, 3]. SCACP often presents as a fleshy or vascular-appearing exophytic tumor with overlying crusting due to the exudative nature of the apocrine glands [4]. These masses can also ulcerate and become painful [5].

Wide local excision is the treatment of choice for SCACP, though there have been cases where SCACP was treated effectively with chemotherapy and radiation in patients with inoperable tumors or who are poor surgical candidates [6]. Specific recommendations regarding surgical margins have not been established. In evaluating data from 49 patients with SCACP, locoregional lymph node metastasis was reported in 22% of patients and distant metastasis in 6% of patients. All patients with disseminated disease in this case series expired from sequelae related to their SCACP diagnosis [1]. Local recurrence with and without distant metastasis has been described [1, 3]. Although SCACP has been traditionally thought to be a low-grade malignancy with a favorable prognosis [7], more recent studies suggest close follow-up is warranted.

Pathologic examination of SCACP resembles SCAP. In both variants, the epithelium is typically composed of an inner columnar cell layer and an outer cuboidal layer. The stroma of the tumor often contains plasma cells and other inflammatory cells [8]. In SCACP, the tumors can extend into underlying tissues and exhibit a high nuclear-to-cytoplasmic ratio, coarse chromatin, and increased mitotic activity [9]. Several of these characteristics were noted in our patient's tumor.

Histologically, SCACP can vary widely in presentation, ranging from an adenocarcinoma-like appearance to having features more consistent with invasive squamous cell carcinoma [10]. This histologic variability can make differentiation of SCACP from other neoplasms arising from adnexal structures challenging [11]. Moreover, distant metastasis from other primary adenocarcinomas can also resemble SCACP [5]. Characteristics that can aid in the diagnosis of SCACP include the continuity with the epidermis and visualization of areas of residual SCAP or sebaceous nevus [10]. Histologic staining, especially with p63 stains and GCDFP-15, can also be useful to aid in diagnosis [11].

The genetic landscape of SCACP is poorly characterized; however, aberrant signaling of the RAS-MAPK pathway has been associated with benign precursors, including nevus sebaceous and SCAP. Mosaicism for *HRAS* p.Gly13Arg and *KRAS* p.Gly12Asp was first described in nevus sebaceous, prompting additional study in secondary tumors arising from this lesion. *BRAF* p.Val600Glu was later identified in a subset of SCAP. In this case report, we expand the genomic landscape to include activating alterations within the *MAP*2*K*1 gene, as the *MAP*2*K*1 p.Leu101_Lys104delinsAsnSer is predicted to activate RAS-MAPK signaling. Given the clinical utility of MEK inhibitors in tumors with described RAS-MAPK pathway activation and ongoing clinical trials, the utility for targeted inhibition in patients with in-frame MEK1 *β*3-*α*C loop alterations has been explored. Notably, allosteric MEK inhibitors (MEKi), such as trametinib, are ATP noncompetitive kinase inhibitors that bind and stabilize an inactive conformation of MEK1. *MAP*2*K*1 missense and in-frame deletions occurring outside of the *β*3-*α*C loop (amino acids 98–104) demonstrate full or partial dependence on RAF activation and exhibit sensitivity to allosteric MEKi. However, in-frame deletions within the *β*3-*α*C loop, such as described in this report, result in RAF-independent stability of the inactive MEK conformation and resistance to allosteric MEKi. A selective ATP-competitive MEKi, MAP855, has shown promising results through inhibition of pERK across all *MAP*2*K*1 alterations and may be of future benefit to patients with tumor harboring in-frame *β*3-*α*C loop deletions.

In conclusion, SCACP is a rare malignancy often arising within an existing nevus sebaceous or SCAP in adults. Our case report demonstrates that SCACP can also rarely affect pediatric patients. Awareness of such atypical presentations can facilitate timely diagnosis. Standard clinical management guidelines for surgical management and surveillance are lacking, and future studies outlining strategies for clinical management are desired.

## Data Availability

No data were used to support this study.

## Supplementary Materials

Supplementary Materials The supplementary materials provide additional details regarding patient enrollment, DNA and RNA sequencing, and Sanger sequencing used in our study, along with additional references pertinent to this section.
