# Supplementary material for: Syringocystadenocarcinoma Papilliferum in a Fifteen-Year-Old Girl: A Case Report and Review of the Literature
Source: Case Rep Dermatol Med. 2022 Feb 3;2022:8076649. doi: 10.1155/2022/8076649 (PMC8831041; doi:10.1155/2022/8076649)
Supplement: Supplementary Materials — The supplementary materials provide additional details regarding patient enrollment, DNA and RNA sequencing, and Sanger sequencing used in our study, along with additional references pertinent to this section. [file 8076649.f1.docx]

**Syringocystadenocarcinoma Papilliferum in a Fifteen-Year-Old Girl:**

**Case Report and Review of the Literature**

**SUPPLEMENTAL MATERIALS AND METHODS**

**Patient enrollment**

The patient was enrolled as part of an Institutional Review Board (IRB) approved study at The Steve and Cindy Rasmussen Institute for Genomic Medicine (IGM) at Nationwide Children’s Hospital (NCH). Informed consent was provided for comprehensive genomic analysis. Peripheral blood was collected by routine venipuncture for genomic DNA extraction. Snap frozen tissue from the tumor was obtained for DNA and RNA extraction.

**DNA sequencing**

Enhanced exome sequencing was performed on DNA extracted from a comparator germline sample (peripheral blood) and disease-involved snap frozen tumor. Libraries were prepared using 100 ng of input DNA beginning with enzymatic fragmentation followed by end repair, 5' phosphorylation, A-tailing, and sequencing adapter ligation using NEB Ultra II FS (New England Biolabs). Target enrichment by hybrid capture was performed with IDT xGen Lockdown v2.0 human exome reagent enhanced with the xGenCNV Backbone Panel and Cancer spike-in (Integrated DNA Technologies, Coralville, IA). Paired-end 151-bp reads were generated on the Illumina NovaSeq (Illumina, Inc., San Diego, CA). Secondary analysis was performed using Churchill, a comprehensive workflow for taking raw reads from alignment through to germline and somatic variants calls^1^. Reads were aligned to the human genome reference sequence (build GRCh38) using BWA (v0.7.15). Sequence alignments were refined according to community-accepted guidelines for best practices (<https://www.broadinstitute.org/gatk/guide/best-practices>). Duplicate sequence reads were removed using samblaster-v.0.1.25, and base quality score recalibration was performed on the aligned sequence data using the Genome Analysis Toolkit (GATK) v4.1.9.0^2^. Germline variants were called using GATK’s HaplotypeCaller^3^. Enhanced exome sequencing average coverage depth was 238X and 196X for the tumor and comparator germline sample, respectively. Somatic single nucleotide variation (SNV) and indel detection was performed using GATK’s MuTect-2^4^. Germline variation in cancer and other disease-associated genes and somatic variation across the coding region of the exome were analyzed^5^. Copy number alteration (CNA) was assessed using both VarScan2^6^ and GATK’s CNA workflow^2,6^. Lollipop plots were generated by MutationMapper using publicly available data from cBioPortal^7,8^.

**RNA-sequencing**

In parallel, a 500 ng aliquot of snap frozen tumor derived RNA was subjected 0.8x SPRI bead cleanup and size selection prior to DNase treatment and ribodepletion prior to using the NEB Next Ultra II Directional kit preparation (performed with 5-minute chemical fragmentation). The library was constructed for whole transcriptome sequencing (RNA-seq). Paired-end 151-bp reads were generated on the Illumina NovaSeq (Illumina, Inc.), and reads were aligned to the human genome reference sequence (GRCh38) with the resultant output representing 69,160,174 uniquely mapped reads. RNA-seq data were processed using an ensemble approach of seven fusion callers (arriba^9^, CICERO^10^, FusionCatcher^11^, FusionMap^12^, JAFFA^13^, MapSplice^14^, and STAR-Fusion^15^) with high-confidence fusions characterized by overlap of fusion identification between the multiple callers. Transcripts per million (TPM) values were generated from paired-end RNA sequence data using Salmon with bootstrapping set to 100^16^.

**Sanger sequencing**

We used 500 ng of RNA with MultiScribe reverse transcriptase (ThermoFisher, Waltham, MA) and random hexamers (Applied Biosystems, Foster City, CA) for RT-PCR. PCR of cDNA was performed with the forward primer in *MAP2K1* exon 3 (5’ GTAAAACGACGGCCAGTTTCATCCCTTCCTCCCTCT 3’) and reverse primer exon 3 (5’ CAGGAAACAGCTATGACGTCACCTCCCAGACCAAAGA 3’). PCR product was purified using the QIAquick purification kit (Qiagen, Germantown, MD). Forward and reverse Sanger sequencing reactions were performed with the Big Dye v3.1 terminator mix (ThermoFisher, Waltham, MA). Sequencing was performed on the Applied Biosystems 3130 instrument (ThermoFisher).

**REFERENCES**

1. Kelly BJ, Fitch JR, Hu Y, et al. Churchill: An ultra-fast, deterministic, highly scalable and balanced parallelization strategy for the discovery of human genetic variation in clinical and population-scale genomics. *Genome Biol.* 2015;16:6. doi:10.1186/s13059-014-0577-x.

2. Van der Auwera G, O’Connor B. *Genomics in the Cloud: Using Docker, GATK, and WDL in Terra (1st Edition)*. O’Reilly Media; 2020.

3. Poplin R, Ruano-Rubio V, DePristo MA, et al. Scaling accurate genetic variant discovery to tens of thousands of samples. *BioRxiv* 2017:201178. doi:10.1101/201178.

4. Cibulskis K, Lawrence MS, Carter SL, et al. Sensitive detection of somatic point mutations in impure and heterogeneous cancer samples. *Nat. Biotechnol.* 2013;31:213–219. doi:10.1038/nbt.2514.

5. Zhang J, Walsh MF, Wu G, et al. Germline Mutations in Predisposition Genes in Pediatric Cancer. *N. Engl. J. Med.* 2015;373:2336–2346. doi:10.1056/NEJMoa1508054.

6. Koboldt DC, Zhang Q, Larson DE, et al. VarScan 2: Somatic mutation and copy number alteration discovery in cancer by exome sequencing. *Genome Res.* 2012;22:568–576. doi:10.1101/gr.129684.111.

7. Cerami E, Gao J, Dogrusoz U, et al. The cBio Cancer Genomics Portal: An open platform for exploring multidimensional cancer genomics data. *Cancer Discov.* 2012;2:401–404. doi:10.1158/2159-8290.CD-12-0095.

8. Gao J, Aksoy BA, Dogrusoz U, et al. Integrative analysis of complex cancer genomics and clinical profiles using the cBioPortal. *Sci. Signal.* 2013;6. doi:10.1126/scisignal.2004088.

9. Uhrig S, Ellermann J, Walther T, et al. Accurate and efficient detection of gene fusions from RNA sequencing data. *Genome Res.* 2021;31. doi:10.1101/gr.257246.119.

10. Tian L, Li Y, Edmonson MN, et al. CICERO: A versatile method for detecting complex and diverse driver fusions using cancer RNA sequencing data. *Genome Biol.* 2020;21. doi:10.1186/s13059-020-02043-x.

11. Nicorici D, Satalan M, Edgren H, et al. *FusionCatcher - a tool for finding somatic fusion genes in paired-end RNA-sequencing data*. Cold Spring Harbor Labs Journals; 2014. doi:10.1101/011650.

12. Ge H, Liu K, Juan T, et al. FusionMap: detecting fusion genes from next-generation sequencing data at base-pair resolution. *Bioinformatics* 2011;27:1922–1928. doi:10.1093/bioinformatics/btr310.

13. Davidson NM, Majewski IJ, Oshlack A. JAFFA: High sensitivity transcriptome-focused fusion gene detection. *Genome Med.* 2015;7:43. doi:10.1186/s13073-015-0167-x.

14. Wang K, Singh D, Zeng Z, et al. MapSplice: Accurate mapping of RNA-seq reads for splice junction discovery. *Nucleic Acids Res.* 2010;38:e178. doi:10.1093/nar/gkq622.

15. Haas BJ, Dobin A, Li B, et al. Accuracy assessment of fusion transcript detection via read-mapping and de novo fusion transcript assembly-based methods. *Genome Biol.* 2019;20:1–16. doi:10.1186/s13059-019-1842-9.

16. Patro R, Duggal G, Love MI, et al. Salmon provides fast and bias-aware quantification of transcript expression. *Nat. Methods* 2017;14:417–419. doi:10.1038/nmeth.4197.
